# An overview of the evidence to guide decision-making in acupuncture therapies for early recovery after acute ischemic stroke

**DOI:** 10.3389/fneur.2022.1005819

**Published:** 2022-10-13

**Authors:** Liuding Wang, Xiansu Chi, Jian Lyu, Zhenmin Xu, Guojing Fu, Yue Liu, Shaojiao Liu, Wenran Qiu, Hongxi Liu, Xiao Liang, Yunling Zhang

**Affiliations:** Xiyuan Hospital, China Academy of Chinese Medical Sciences, Beijing, China

**Keywords:** acupuncture, acute ischemic stroke, neurological function, GRADE, AMSTAR-2

## Abstract

**Background:**

Acupuncture is a proven technique of traditional Chinese medicine (TCM) for ischemic stroke. The purpose of this overview was to summarize and evaluate the evidence from current systematic reviews (SRs) of acupuncture for early recovery after acute ischemic stroke (AIS).

**Methods:**

We performed a comprehensive search for SRs of acupuncture for AIS in seven electronic databases up to May 23, 2022. Two reviewers independently selected SRs, extracted data, evaluated the methodological quality using the Assessment of Multiple Systematic Reviews 2 (AMSTAR 2), and rated evidence certainty using the Grading of Recommendations, Assessment, Development, and Evaluation (GRADE).

**Results:**

Seven SRs were included. The overall methodological quality of SRs was critically low. As for GRADE, 3 outcomes had moderate-quality evidence, 14 had low-quality evidence, and 12 had very low-quality evidence. Moderate-quality evidence demonstrated that initiating acupuncture therapies within 30 days of AIS onset significantly improves neurological function and the total effective rate of patients. Low-quality evidence showed that for patients within 2 weeks of AIS onset Xingnao Kaiqiao acupuncture (XNKQ Ac) could reduce disability rate and might reduce mortality. Regarding the safety of acupuncture therapies, low-quality evidence showed that there was no difference in the incidence of adverse reactions between the 2 groups, and very-low quality evidence showed that acupuncture did not promote hemorrhagic conversion.

**Conclusions:**

In the acute and early recovery phases after AIS onset, acupuncture is a promising therapeutic strategy to improve the curative effect of current treatments, especially in the recovery of neurological function. Patients in the acute phase might receive XNKQ Ac, and patients in the early recovery phase might receive EA^1^, CA, or SA. However, considering the current certainty of evidence, a solid recommendation warrants further exploration.

**Systematic review registration:** https://www.crd.york.ac.uk/PROSPERO/, identifier CRD42022335426.

## Introduction

At present, acute ischemic stroke (AIS) remains a prominent cause of death and disability worldwide (1), despite breakthroughs in emergency therapy over the past years. Intravenous thrombolysis (IVT) and endovascular therapies (EVTs) have been the preferred treatments for patients with AIS (2). However, the majority of AIS patients did not receive IVT or EVTs due to late arrival to emergency departments. As for patients fortunately treated with recanalization therapies, they may suffer from ischemia/reperfusion (I/R) injury caused by highly harmful oxidative stress (OS) (3). In other words, even with currently evidence-based, effective therapies, there is a lack of an optimum therapeutic strategy to timely protect the brain from damage in the acute or early recovery stages.

As one of the various modalities of traditional Chinese medicine (TCM), acupuncture has gained international recognition, particularly in recent years (4). In the treatment of ischemic stroke, previous studies have indicated that acupuncture might prevent secondary brain injury by reducing oxidation (5). This potential mechanism of acupuncture removing superoxide has also been demonstrated in other nervous system diseases, such as vascular dementia and spinal cord injury (6–8). In animal models of ischemic stroke, acupuncture therapies may not only suppress the excessive production of reactive oxygen species (ROS), but also activate the inherent antioxidant enzymes (9). Fundamentally, acupuncture therapies may ameliorate mitochondrial dysfunction, which is manifested in raising the activities of mitochondrial respiratory enzymes (10). Regarding clinical benefits, there were numerous systematic reviews (SRs) evaluating the efficacy of acupuncture therapies for ischemic stroke from acute to convalescent and sequela stages (11–14). Since oxidative damage is the most severe within 24 h after onset (15), it is of great significance to investigate the efficacy and safety of acupuncture for ischemic stroke during the acute stage. A network meta-analysis also showed that the optimal time-point of acupuncture for stroke was within 48 h post-stroke, and the vital validity period lasted until 15 days after the attack (16). However, the paucity of systematic evaluation of evidence certainty is the reason why acupuncture therapies cannot be brought to the bedside of patients with AIS. The overview of SRs has been generally recognized to facilitate clinical decision-making. A study published in the *British Medical Journal* strongly calls for more effective evidence dissemination of acupuncture to solve the dilemma that evidence on acupuncture is underused in clinical practice (4). Therefore, we conducted this overview to summarize the existing evidence and critically evaluate the overall evidence quality of acupuncture therapies for early recovery after AIS.

## Methods

This overview was carried out according to the Cochrane Handbook for SRs of Interventions (17) and registered under the number CRD42022335426.

### Inclusion and exclusion criteria

We included SRs of randomized controlled trials (RCTs), in which participants were diagnosed with AIS and in the acute and early recovery phases (within 1 month after AIS onset). Acupuncture therapies were used alone or combined with conventional therapy (CT, including recanalization treatments, controlling vascular risk factors, improving blood circulation, and protecting brain cells). Comparator interventions were CT alone, or CT combined with placebo or sham acupuncture. Outcomes included but were not limited to functional independence (modified Rankin Scale score 0–2), mortality, disability rate, neurologic deficit score (NDS), activities of daily living (ADL), and adverse reactions.

We excluded studies if they were repeated publications; if their full text were unavailable; if they had incomplete or inaccurate data; if they used other TCM treatments in either intervention or control group.

### Search strategy

We searched seven electronic databases listed below from their inception to 23 May 2022: MEDLINE Ovid (1946 to 23 May 2022), EMBASE Ovid (1996 to 2022 Week 20), the Cochrane Library, Chinese Biomedical Literature Service System (SinoMed), China National Knowledge Infrastructure (CNKI), Chinese Scientific Journals Database (VIP), and WanFang database. The search strategies for all databases are shown in Supplementary Table 1.

### Study selection and data extraction

Two independent authors (LDW and ZMX) screened the records yielded in searches by reading titles and abstracts. Full texts of preliminary included SRs were further checked, and finally, eligible SRs were identified. And then they used a standard form extracting the following data: (1) first author, country, and publication year; (2) the number of trials and participants, eligibility criteria, interventions, comparisons, outcomes, and conclusions. Disagreements were resolved by consensus.

### Quality assessment

#### Methodological quality of included reviews

Two independent reviewers (XSC and GJF) used the Assessment of Multiple Systematic Reviews 2 (AMSTAR 2) to evaluate the methodological quality of SRs (18). Each of the 16 items was evaluated as “yes,” “partial yes,” or “no.” Items 2, 4, 7, 9, 11, and 13 were regarded as critical. The overall quality was rated as “high (no or 1 non-critical weakness),” “moderate (more than 1 non-critical weakness),” “low (1 critical flaw with or without non-critical weaknesses),” or “critically low (more than 1 critical flaw with or without non-critical weaknesses).” Disagreements were resolved by an expert in methodology (JL). We summarized these results and identified common methodological deficiencies.

#### Evidence certainty of included reviews

We used the Grading of Recommendation, Assessment, Development, and Evaluation (GRADE) system to rate the certainty of evidence as “high,” “moderate,” “low” or “very low.” Certainty was downgraded for five factors (risk of bias, heterogeneity, detected publication bias, imprecision, and indirectness). If possible, we used the GRADEpro “Summary of findings” tables obtained from each included review. Two independent reviewers (WRQ and HXL) performed the evaluation. In cases of any disagreements, we consulted with an expert in methodology (JL).

## Results

### Literature search

**Figure 1:**
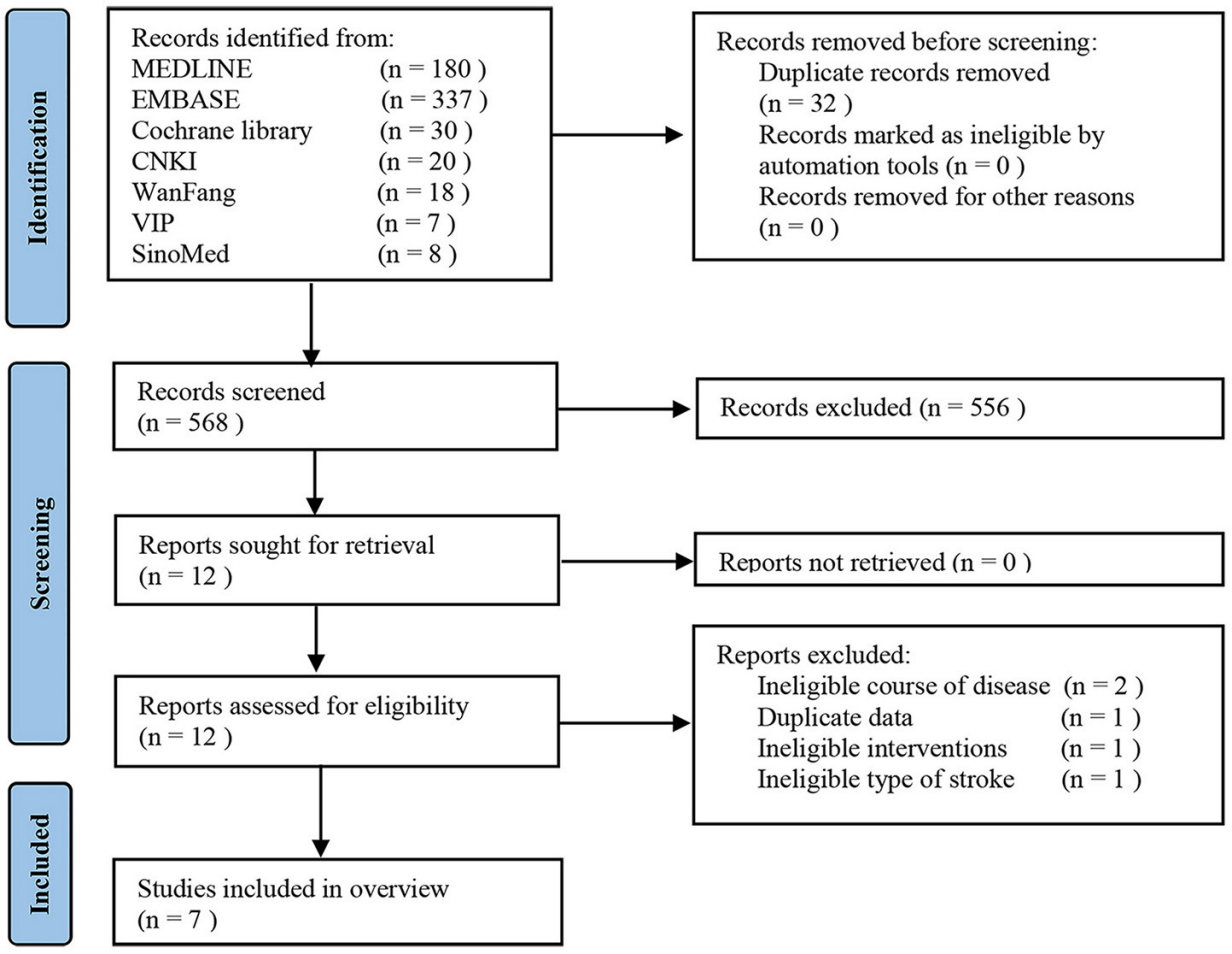
Flow diagram for identification of studies.

A total of 600 records were retrieved. After removing 32 duplicates, we screened the titles and the abstracts of 556 studies, and then assessed the full texts of 12 studies. A list of 5 studies, that appeared to meet the eligibility criteria but were excluded, is shown in Supplementary Table 2 along with reasons for exclusion. Ultimately, 7 SRs met the eligibility criteria (19–25). Figure 1 shows details of selecting studies.

### Characteristics of included reviews

Table 1 presents a summary of included SRs. Original trials were published between 1996 and 2020. The date of the last retrieval in the SRs varied between October 2007 and December 2020. Four SRs were in the English language (19–22), and the other 3 were Chinese (23–25). The number of participants varied across SRs, ranging from 429 to 3,792. In total, 114 RCTs involving 9921 participants were included in 7 SRs. The time to initiate acupuncture therapies in patients was within 48 h of AIS onset in 1 SR (23), 14 days in 2 SRs (20, 22), and 1 month in 4 SRs (19, 21, 24, 25). Acupuncture therapies included conventional acupuncture (CA), electroacupuncture (EA^1^), eye acupuncture (EA^2^), foot acupuncture (FA), scalp acupuncture (SA), governor vessel acupuncture (GV Ac), and Xingnao Kaiqiao needling method acupuncture (XNKQ Ac). SRs reported outcomes: mortality, disability rate, modified Rankin Scale (mRS), NDS, ADL, motor impairment, total effective rate, complete recanalization, hemorrhagic conversion, C-reaction protein (CRP), adverse reactions, and adverse events. The measurements of NDS included the National Institute of Health Stroke Scale (NIHSS), the Chinese Stroke Scale/Modified Edinburgh-Scandinavia Stroke Scale (CSS/MESSS), and the Scandinavia Stroke Scale (SSS). ADL was assessed using the Barthel Index (BI). Motor impairment was measured with the Fugl–Meyer Assessment (FMA). Most SRs concluded that acupuncture is effective in treating AIS. Nevertheless, the paucity of high-quality RCTs downgraded the certainty of evidence.

**Table 1:** Characteristics of included SRs.

**Reference**	**Language**	**No. of studies (participants)**	**Course of disease**	**Treatment intervention vs. control intervention**	**Quality assessment tool**	**Outcome (s)**	**Main conclusion**
Shao et al. (19)	English	18 (1,543)	<30 days	GV Ac + CT vs. CT; GV Ac + CT vs. CA + CT	the Cochrane risk of bias tool	Neurological deficit score, activities of daily living, functional independence, adverse events	GV Ac combined with CT may increase the benefit, and for efficacy GV Ac appears to be better than that of ordinary acupuncture.
Yang et al. (20)	English	12 (1,006)	<14 days	XNKQ Ac + CT vs. CT; XNKQ Ac + CA vs. CA	the Cochrane risk of bias tool	Mortality, disability rate, activities of daily living, total effective rate, adverse events	XNKQ Ac might significantly reduce the disability rate, but it had no significant impact on mortality. XNKQ Ac is effective and safe for AIS, but more high-quality randomized controlled trials are needed to provide reliable evidence.
Liu et al. (21)	English	18 (1,411)	<30 days	EA^1^ + CT vs. CT	the Cochrane risk of bias tool	Neurological deficit score, activities of daily living, motor function, total effective rate, adverse events	EA was effective and generally safe for AIS, although further larger sample-size and rigorously designed RCTs are required.
Wang et al. (22)	English	8 (538)	<14 days	SA + CT vs. CT	the Cochrane risk of bias tool	Neurological deficit score, total effective rate, adverse events	SA appears to be able to improve neurological deficit score and the clinical effective rate, though the beneficial effect from SA is possibly overvalued because of generally low methodology of the included trials. Rigorous well-designed clinical trials are needed.
Zhang et al. (23)	Chinese	14 (1,202)	≤48 h	XNKQ Ac, EA^2^, FA, or CA + thrombolysis + CT vs. thrombolysis + CT	the Cochrane risk of bias tool	Neurological deficit score, activities of daily living, total effective rate, C-reaction protein level, the rate of complete recanalization, hemorrhagic conversions, incidence of adverse reactions	Acupuncture has certain advantages in improving the therapeutic effect and safety of thrombolysis in the treatment of AIS.
Zhang and Li (24)	Chinese	39 (3,792)	≤30 days	CA, EA^1^, EA^2^, SA, XNKQ Ac, or A&M + CT vs. CT	NOS scale	Neurological deficit score, total effective rate	Acupuncture plus CT is effective in treating AIS, but more randomized, double-blind, large-sample trials are needed.
Zhang et al. (25)	Chinese	5 (429)	<28 days	CA or EA^1^ + CT vs. CT	/	Mortality, disability rate, neurological deficit score	The result cannot prove that acupuncture can set down disability and mortality. Although acupuncture can improve the neurological function of AIS patients, we cannot sure the curative effect. We need randomized, double blinded, controlled trials with high-quality, large-sample, multi-center to get believable evidence.

Ac, acupuncture; A&M, acupuncture and moxibustion; CA, conventional acupuncture; CT, conventional therapy; EA^1^, electroacupuncture; EA^2^, eye acupuncture; FA, foot acupuncture; GV, governor vessel; SA, scalp acupuncture; XNKQ, Xingnao Kaiqiao needling method.

### Efficacy of acupuncture for AIS

Results of all efficacy outcomes are presented in tabular form (Supplementary Table 3).

#### NDS

##### Acupuncture + conventional therapy vs. conventional therapy

Four SRs adopted NIHSS measuring NDS and revealed that acupuncture therapies were associated with a significant reduction in NIHSS (19, 21, 23, 24). The heterogeneity of 2 SRs was insignificant (4 RCTs, *MD* = −1.18, 95% *CI*−1.52 to−0.83, *P* < 0.00001, *I*^2^ = 0%; 17 RCTs, *MD* = −1.86, 95% *CI*−2.06 to−1.66, *P* < 0.00001, *I*^2^ = 22%) (19, 24). The other SRs had significant heterogeneity (6 RCTs, *SMD* = −0.81, 95% *CI*−1.14 to−0.49, *P* < 0.00001, *I*^2^ = 51%; 12 RCTs, *MD* = −3.51, 95% *CI*−4.54 to−2.48, *P* < 0.00001, *I*^2^ = 90%).

Three SRs used CSS/MESSS to assess NDS and revealed that acupuncture therapies were associated with a significant reduction in CSS/MESSS (19, 21, 22). The heterogeneity of 1 SR was insignificant (3 RCTs, *MD* = −3.77, 95% *CI*−4.98 to−2.57, *P* < 0.00001, *I*^2^ = 0%) (19), and that of the other SRs was significant (5 RCTs, *SMD* = −1.27, 95% *CI*−2.18 to−0.37, *P* = 0.006, *I*^2^ = 94%; 7 RCTs, *MD* = −3.89, 95% *CI* −5.36 to −2.43, *P* < 0.00001, *I*^2^ = 57%).

One SR used SSS to assess NDS and reported that SSS in the acupuncture group was higher than that in the control group with statistical significance (25), but the heterogeneity was significant (3 RCTs, *MD* = 3.49, 95% *CI* 2.00 to 4.99, *P* < 0.00001, *I*^2^ =81.9%).

##### Governor vessel acupuncture vs. conventional acupuncture

One SR reported that NIHSS in the GV Ac group was lower than that in the CA group with statistical significance, but the heterogeneity was significant (2 RCTs, *MD* = −1.32, 95% *CI*−2.18 to−0.47, *P* = 0.002, *I*^2^ = 75%) (19).

One SR reported that CSS/MESSS in the GV Ac group was lower than that in the CA group with statistical significance (19), but the heterogeneity was significant (3 RCTs, *MD* = −4.63, 95% *CI*−5.91 to−3.35, *P* < 0.00001, *I*^2^ = 50%).

#### BI

##### Acupuncture + conventional therapy vs. conventional therapy

Three SRs showed that the acupuncture group was superior to the control group in improving BI with statistical significance. In these 3 SRs, 1 SR performed a subgroup analysis according to the course of treatment (2 RCTs, *MD*_≤15d_ = 22.55, 95% *CI* 18.66 to 26.45, *P* < 0.00001, *I*^2^ = 0%; 3 RCTs, *MD*_>15d_ = 8.80, 95% *CI* 5.87 to 11.72, *P* < 0.0001, *I*^2^ = 0%) (19). A subgroup analysis according to thrombolytic drugs was performed in another SR (23), which failed to explain the significant heterogeneity.

##### Governor vessel acupuncture vs. conventional acupuncture

One SR reported that BI in the GV Ac group was higher than that in the CA group with statistical significance (19), but the heterogeneity was significant (5 RCTs, *MD* = 8.27, 95% *CI* 4.29 to 12.26, *P* < 0.0001, *I*^2^ = 78%).

##### Xingnao kaiqiao needling method acupuncture + conventional acupuncture vs. conventional acupuncture

One SR reported that XNKQ Ac plus CA improved BI better than CA alone (1 RCT, *MD* = 17.17, 95% *CI* 9.15 to 25.19, *P* < 0.0001) (20).

#### mRS

##### Acupuncture + conventional therapy vs. conventional therapy

One SR reported that mRS at 1 month in the acupuncture group was lower than that in the control group (3 RCTs, *MD* = −0.63, 95% *CI*−0.95 to−0.32, *P* < 0.0001, *I*^2^ = 0%) (19). Although the difference had statistical significance, the effect size was far <1 point. As for the evaluation of mRS, 1 point is required per grade. In terms of clinical benefits, therefore, the clinical significance of the difference was insignificant.

#### FMA

##### Acupuncture + conventional therapy vs. conventional therapy

One SR reported that FMA in the acupuncture group was higher than that in the control group with statistical significance (8 RCTs, *SMD* = 0.98, 95% *CI* 0.75 to 1.22, *P* < 0.00001, *I*^2^ = 36%) (21).

#### Mortality and disability rate

##### Xingnao kaiqiao acupuncture + conventional acupuncture vs. conventional acupuncture

After 3 or 6 months of follow-up, 1 SR reported that XNKQ Ac plus CA might have additional effects in reducing disability rate with statistical significance and mortality without statistical significance (3 RCTs, *RR*_disability_ = 0.51, 95% *CI* 0.27 to 0.98, *P* = 0.04, *I*^2^ = 0%; 3 RCTs, *RR*_mortality_ = 0.58, 95% *CI* 0.17 to 1.93, *P* = 0.37, *I*^2^ = 0%) (20). The confidence interval of disability rate was so wide that it was close to the invalid line, and the confidence interval of mortality even included the invalid line. Given the imprecision, the reliability of the results is questionable.

##### Acupuncture + conventional therapy vs. conventional therapy

One SR defined disabled as BI ≤ 60 (25), and reported that there was no statistical difference in the rate of death or disability at 6 months of follow-up between the acupuncture group and the control group. However, there was a trend toward reduced rate of death or disability with additional acupuncture therapies (3 RCTs, *OR* = 0.59, 95% *CI* 0.31 to 1.13, *P* = 0.11, *I*^2^ = 0%).

#### Total effective rate

##### Acupuncture + conventional therapy vs. conventional therapy

Four SRs reported that acupuncture might improve total effective rate with insignificant heterogeneity (6 RCTs, *RR* = 1.42, 95% *CI* 1.18 to 1.72, *P* = 0.0002, *I*^2^ = 16%; 4 RCT, *RR* = 1.23, 95% *CI* 1.11 to 1.37, *P* < 0.01, *I*^2^ = 0%; 13RCTs, *RR* = 1.19, 95% *CI* 1.13 to 1.25, *P* < 0.00001, *I*^2^ = 41%; 26 RCTs, *OR* = 3.95, 95% *CI* 3.02 to 5.16, *P* < 0.00001, *I*^2^ = 0%) (21–24). Another SR performed a subgroup analysis of different courses of disease (2 RCTs, *RR*_≤24h_ = 1.40, 95% *CI* 1.06 to 1.86, *P* = 0.02, *I*^2^ = 2%; 2 RCT, *RR*_6h − 72h_ = 1.63, 95% *CI* 1.03 to 2.59, *P* = 0.04, *I*^2^ = 45%) (20).

##### Xingnao kaiqiao acupuncture + conventional acupuncture vs. conventional acupuncture

One SR reported that the total effective rate in the XNKQ Ac plus CA group was higher than that in the CA group without statistical significance (1 RCT, *RR* = 1.80, 95% *CI* 1.00 to 3.23, *P* = 0.05) (20).

#### Rate of complete recanalization

##### Acupuncture + conventional therapy vs. conventional therapy

One SR found that the use of acupuncture after thrombolysis might improve the rate of complete recanalization without statistical significance (2 RCTs, *RR* = 1.20, 95% *CI* 1.00–1.44, *P* = 0.05, *I*^2^ = 0%) (23).

#### CRP

##### Acupuncture + conventional therapy vs. conventional therapy

One SR revealed that CRP in the acupuncture group was lower than that in the control group with statistical significance and insignificant heterogeneity (2 RCTs, *MD* = −3.99, 95% *CI*−4.35 to−3.63, *P* < 0.00001, *I*^2^ = 0%) (23).

### Safety of acupuncture for AIS

Two SRs reported no adverse events (20, 22), and 3 SRs reported adverse events in detail (19, 21, 23). The adverse events in the acupuncture group mainly included subcutaneous hematoma, ecchymosis, and needle stagnation. One SR reported that the hemorrhagic conversion rate in the acupuncture group was lower than that in the control group without statistical significance (3 RCTs, *RR* = 0.72, 95% *CI* 0.14–3.62, *P* = 0.69, *I*^2^ = 51%) (23).

### Quality assessment

#### Methodological quality of included reviews

**Figure 2:**
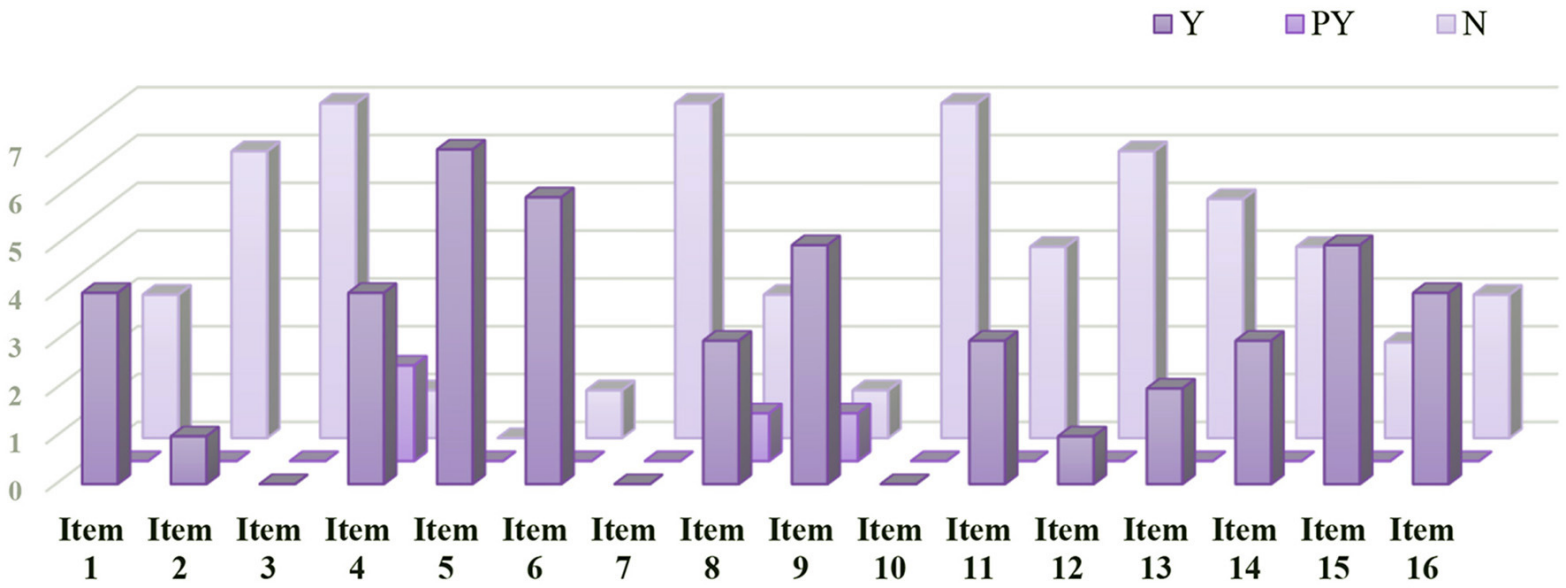
Methodological quality assessment by AMSTAR 2.

We summarized the methodological quality of included SRs in Table 2 and Figure 2. Seven critical items were poorly reported. Just 1 SR provided a well-developed protocol (item 2) (19). Three SRs only searched electronic databases, but not clinical registries or gray literature (item 4) (22–24). None of the SRs presented a list of each excluded trial with specific reasons (item 7). Two SRs used an inappropriate technique to assess the risk of bias (RoB) (item 9) (24, 25). More than half of SRs lacked an exploration of substantial heterogeneity (item 11) (21, 22, 24, 25). And most SRs did not consider the impact of RoB when discussing their results (item 13) (19, 20, 22–24). One SR carried out a quantitative analysis, the Egger test, to detect the publication bias (19), 4 SRs performed a qualitative analysis (21–24), and 2 SRs did not mention it (item 15) (20, 25). As for 9 non-critical items, the poorly reported ones mainly were the reason for selecting RCTs (item 3), the sources of funding (item 10), and the assessment of the potential impact of RoB on meta-analysis (item 12).

**Table 2:** Methodological quality assessment by AMSTAR 2.

**Reviews**	**AMSTAR-2**																**Total yes**	**Overall quality**
	**Item 1**	**Item 2**	**Item 3**	**Item 4**	**Item 5**	**Item 6**	**Item 7**	**Item 8**	**Item 9**	**Item 10**	**Item 11**	**Item 12**	**Item 13**	**Item 14**	**Item 15**	**Item 16**		
Shao et al. (19)	Y	Y	N	Y	Y	Y	N	PY	Y	N	Y	N	N	Y	Y	Y	10	Critically low
Yang et al. (20)	N	N	N	Y	Y	Y	N	Y	Y	N	Y	N	N	Y	N	Y	9	Critically low
Liu et al. (21)	Y	N	N	Y	Y	Y	N	Y	Y	N	N	Y	Y	N	Y	N	9	Critically low
Wang et al. (22)	Y	N	N	N	Y	Y	N	Y	Y	N	N	N	N	N	Y	Y	7	Critically low
Zhang et al. (23)	Y	N	N	PY	Y	Y	N	N	Y	N	Y	N	N	Y	Y	N	7	Critically low
Zhang and Li (24)	N	N	N	PY	Y	N	N	N	N	N	N	N	N	N	Y	N	2	Critically low
Zhang et al. (25)	N	N	N	Y	Y	Y	N	N	PY	N	N	N	Y	N	N	Y	5	Critically low
In total of “Y”	57.14%	14.29%	0	57.14%	100 %	85.71%	0	42.86%	71.43%	0	42.86%	14.29%	28.57%	42.86%	71.43%	57.14%		

Y, yes; PY, partial yes; N, no; Item 1, whether the research question and inclusion criteria included the components of PICO; Item 2, whether to establish the methods prior to the implementation, and whether to report significant deviations from the protocol; Item 3, whether the authors explained the selection of the study designs for inclusion; Item 4, whether the authors used a comprehensive literature search strategy; Item 5, whether the literature screening was performed in duplicate; Item 6, whether the data extraction was performed in duplicate; Item 7, whether a list of excluded studies and reasons for the exclusions were provided; Item 8, whether the authors describe the included studies in detail; Item 9, whether the authors used appropriate methods to assess the risk of bias in included studies; Item 10, whether the authors reported funding; Item 11, if meta-analysis was performed, did the authors use right methods to pool the results; Item 12, if meta-analysis was performed, did the authors consider the impact of risk of bias on the pooled results or other evidence integration; Item 13, whether the authors considered the risk of bias when interpreting the results; Item 14, whether the authors explained any heterogeneity across the review; Item 15, if quantitative synthesis was performed, did the authors detect publication bias and discuss the potential impact on the findings; Item 16, whether the authors reported any conflicts of interest.

#### Evidence certainty of included reviews

One SR evaluated the certainty of evidence (21). Considering objectivity and impartiality, we used the GRADE approach to reevaluate important outcomes of all SRs. We summarized the overall certainty of evidence in Table 3. Ratings ranged from very low to moderate. Evidence of three outcomes was moderate-quality, 14 was low-quality, and 12 was very low-quality. The main reasons for downgrading the certainty across SRs were a high risk of bias (inadequate reporting of randomization, lack of blinding) (100 %), imprecision (79.31 %), and inconsistency (48.28 %).

**Table 3:** Certainty of evidence in included SRs by GRADE.

**Reviews**	**Interventions**	**Outcomes**	**No. of RCT (participant intervention/ control group)**	**Certainty assessment**					**Overall certainty**
				**Risk of bias**	**Inconsistency**	**Indirectness**	**Imprecision**	**Publication bias**	
Shao et al. (19)	GV Ac + CT vs. CT	BI	5 (155/154)	Serious^a^	Serious^b^	Not serious	Serious^c^	Undetected	Very low
		mRS	3 (73/74)	Serious^a^	Not serious	Not serious	Serious^c^	Undetected	Low
		NIHSS	4 (103/104)	Serious^a^	Not serious	Not serious	Serious^c^	Undetected	Low
		CSS/MESSS	3 (94/93)	Serious^a^	Not serious	Not serious	Serious^c^	Undetected	Low
	GV Ac vs. CA	BI	5 (187/181)	Serious^a^	Serious^b^	Not serious	Serious^c^	Undetected	Very low
		NIHSS	2 (58/57)	Serious^a^	Serious^b^	Not serious	Serious^c^	Undetected	Very low
		CSS/MESSS	3 (127/122)	Serious^a^	Serious^b^	Not serious	Serious^c^	Undetected	Very low
Zhang et al. (23)	XNKQ Ac, EA^2^, FA or CA + IVT vs. IVT	Total effective rate	13 (574/568)	Serious^a^	Not serious	Not serious	Not serious	Undetected	Moderate
		NIHSS	12 (509/501)	Serious^a^	Serious^b^	Not serious	Not serious	Undetected	Low
		BI	8 (345/345)	Serious^a^	Serious^b^	Not serious	Not serious	Undetected	Low
		CRP	2 (65/65)	Serious^a^	Not serious	Not serious	Serious^c^	Undetected	Low
		Incidence of adverse reaction	5 (228/228)	Serious^a^	Not serious	Not serious	Serious^c^	Undetected	Low
		Hemorrhagic conversion rate	3 (132/132)	Serious^a^	Serious^b^	Not serious	Serious^c^	Undetected	Very low
Zhang and Li (24)	CA, EA^1^, EA^2^, SA or A&M + CT vs. CT	NIHSS	17 (740/715)	Serious^a^	Not serious	Not serious	Not serious	Undetected	Moderate
		Total effective rate	26 (1,497/1,460)	Serious^a^	Not serious	Not serious	Not serious	Undetected	Moderate
Yang et al. (20)	XNKQ Ac + CT vs. CT	Disability rate	2 (63/65)	Serious^a^	Not serious	Not serious	Serious^c^	Undetected	Low
		Mortality	3 (178/182)	Serious^a^	Not serious	Not serious	Serious^c^	Undetected	Low
		BI	3 (102/104)	Serious^a^	Serious^b^	Not serious	Serious^c^	Undetected	Very low
		Total effective rate	8 (399/402)	Serious^a^	Serious^b^	Not serious	Serious^c^	Undetected	Very low
	XNKQ Ac + CA vs. CA	BI	1 (30/30)	Serious^a^	-	Not serious	Serious^c^	Undetected	Very low
Liu et al. (21)	EA^1^ + CT vs. CT	BI	10 (381/361)	Serious^a^	Serious^b^	Not serious	Not serious	Undetected	Low
		FMA	8 (252/247)	Serious^a^	Not serious	Not serious	Serious^c^	Undetected	Low
		NIHSS	6 (177/168)	Serious^a^	Serious^b^	Not serious	Serious^c^	Undetected	Very low
		CSS	5 (203/194)	Serious^a^	Serious^b^	Not serious	Serious^c^	Undetected	Very low
		Total effective rate	6 (200/195)	Serious^a^	Not serious	Not serious	Serious^c^	Undetected	Low
Wang et al. (22)	SA + CT vs. CT	MESSS	7 (223/213)	Serious^a^	Serious^b^	Not serious	Serious^c^	Undetected	Very low
		Total effective rate	4 (153/155)	Serious^a^	Not serious	Not serious	Serious^c^	Undetected	Low
Zhang et al. (25)	CA or EA^1^ + CT vs. CT	Rate of death or disability	3 (110/115)	Serious^a^	Not serious	Not serious	Serious^c^	Undetected	Low
		SSS	3 (150/135)	Serious^a^	Serious^b^	Not serious	Serious^c^	Undetected	Very low

Ac, acupuncture; A&M, acupuncture and moxibustion; CA, conventional acupuncture; CT, conventional therapy; EA^1^, electroacupuncture; EA^2^, eye acupuncture; FA, foot acupuncture; GV, governor vessel; IVT, intravenous thrombolysis; SA, scalp acupuncture; XNKQ, Xingnao Kaiqiao needling method.

a High risk of bias in allocation concealment and blinding.

b The statistical heterogeneity was significant (I^2^ > 50%).

c Small sample size or too wide confidence interval.

### Subgroup analysis

**Figure 3:**
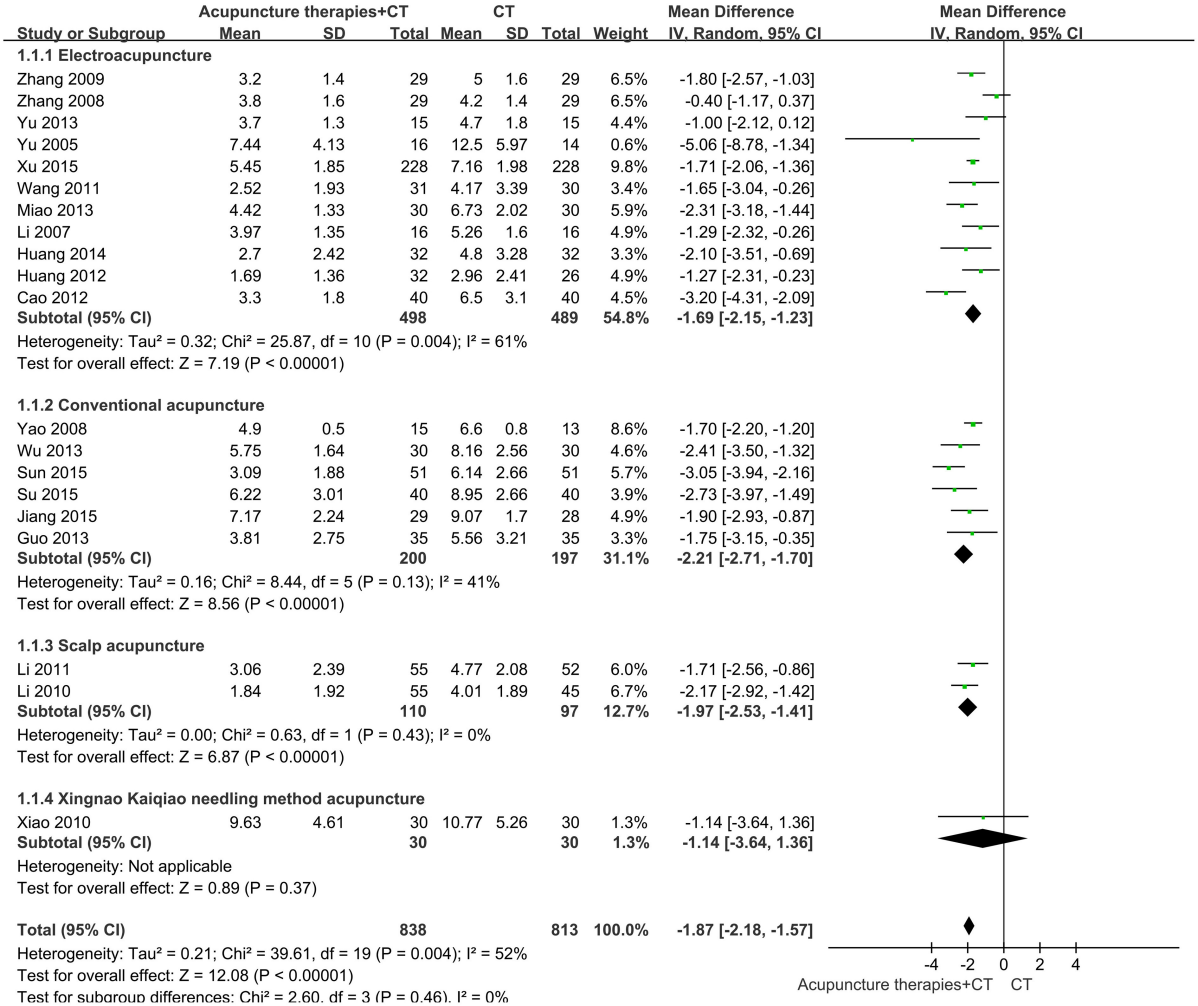
Subgroup analysis of the NIHSS in patients receiving acupuncture therapies within 1 month after AIS onset.

**Figure 4:**
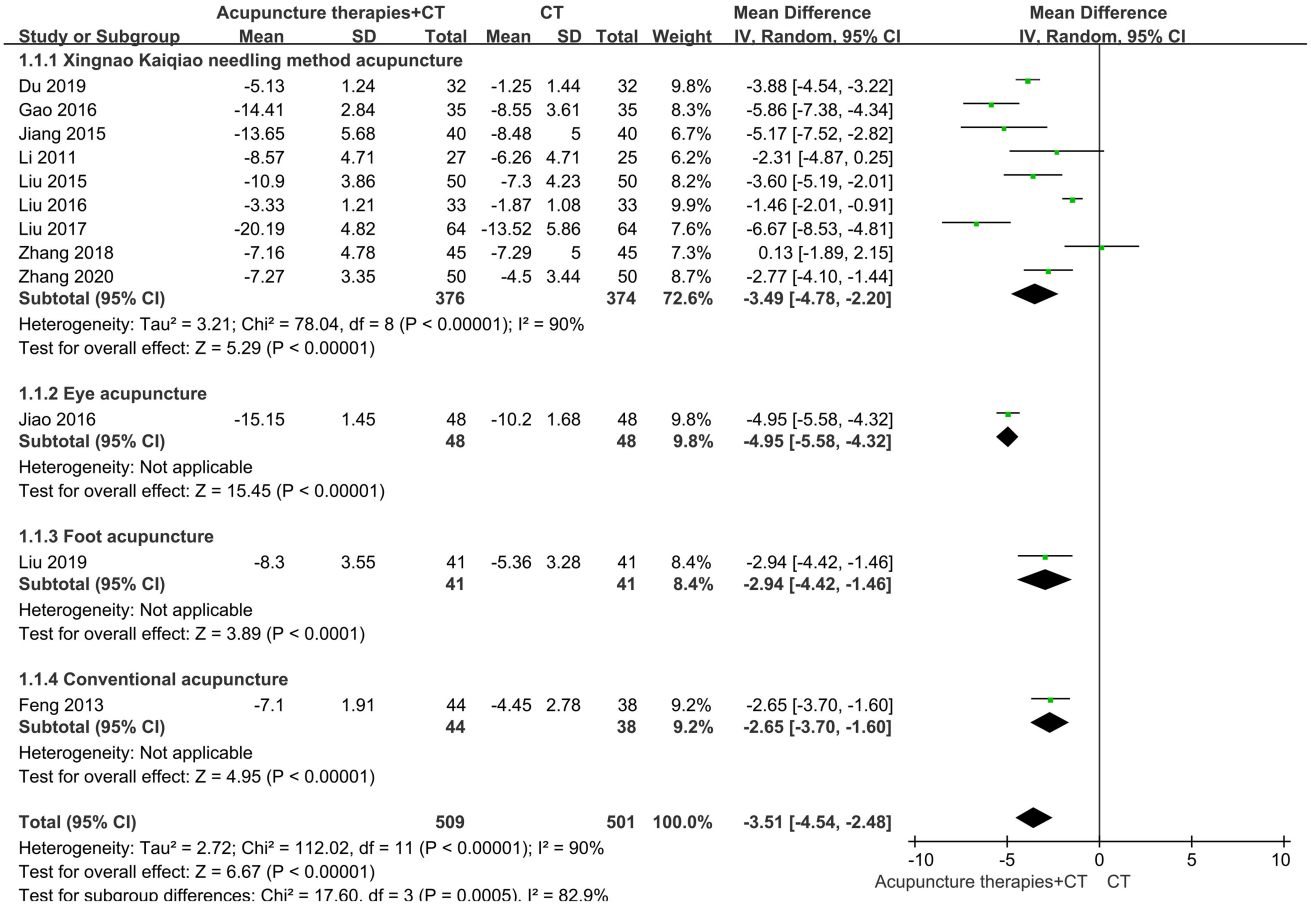
Subgroup analysis of the NIHSS in patients receiving acupuncture therapies within 48 h after AIS onset.

Providing practical guidance in clinical decision-making, we further performed subgroup analysis. As for the NIHSS in patients within 1 month after AIS, we collected original data from 2 SRs (21, 24), pooled these results, and performed subgroup analysis according to acupuncture methods. The results demonstrated that EA^1^, CA, and SA all significantly promoted early recovery of neurological function after AIS onset (Figure 3). The confidence intervals of the 3 subgroups were narrow and statistical heterogeneity was acceptable, indicating that the evidence was reliable. As for the NIHSS in patients within 48 hours after AIS onset, we collected original data from the SR (23) and performed subgroup analysis according to acupuncture methods. The results of all subgroups were statistically significant (Figure 4). But the larger sample size (750 participants) demonstrated that XNKQ Ac was the preferred acupuncture method for patients within 48 hours after AIS onset compared with other acupuncture methods, such as EA^2^, FA, and CA.

## Discussion

The SR of RCTs is ranked as the most rigorous tool for clinical decision-making. Nevertheless, the uneven quality of original RCTs and the non-standard implementation of SR affected the certainty of evidence. A growing number of SRs of acupuncture for various diseases with positive results have been published. Therefore, an overview of SRs is of great significance (26). To our knowledge, this study is the first overview of SRs regarding acupuncture for AIS. In our overview, included SRs provided invaluable evidence on acupuncture therapies for AIS treatment, but many limitations do exist.

### Summary of findings

We identified 7 SRs with 114 RCTs (9,921 participants). Overall methodological quality assessed by AMSTAR 2 was low, and most critical items were reported poorly, particularly in establishing an explicit statement before the conduct of the study, listing the excluded trials with precise explanation, performing sensitivity or subgroup analyses for substantial heterogeneity, and fully assessing the impact of RoB on results. Due to the high risk of bias, significant heterogeneity, and small samples, the level of evidence was correspondingly downgraded. The certainty of evidence was moderate to very low.

Moderate-quality evidence indicated that acupuncture therapies improve neurological function and the total effective rate of patients within 30 days of AIS onset. Several experiments studying rats with middle cerebral artery occlusion demonstrated that acupuncture therapies might ameliorate neurological function by regulating the opening of large-conductance Ca2+-activated potassium channels (27), and repressing ER stress-mediated autophagy and apoptosis (28).

Low-quality evidence suggested that initiating XNKQ Ac within 2 weeks of AIS onset might reduce the disability rate and mortality. Although there was no statistical difference in mortality, the trend of significantly reducing mortality deserves more research. XNKQ Ac was developed by Professor Shi Xuemin, academician of the Chinese Academy of Engineering, in 1972. Two decades ago, the effects of inhibiting free radicals and increasing superoxide dismutase (SOD) were verified in the rabbit model of acute I/R (29). There was an RCT conducted in Germany suggesting the modulation of functional connectivity in areas of motor function by XNKQ Ac (30). A recent clinical trial, comparing XNKQ Ac plus alteplase and alteplase alone, suggested that XNKQ Ac might promote the recovery of neurological function by improving lipid peroxidation (31). Lately, an RCT conducted by Shanghai Jiao Tong University found that the effects of XNKQ Ac may be associated with modulating brain rhythm oscillations of AIS patients (32).

Low-quality evidence demonstrated that the initiation of GV Ac within 3 days of AIS onset improves mRS at 1 month. The measurement of functional independence using mRS is generally considered the primary outcome (33). However, the effect size of mRS reduced by GV Ac was only 0.63, far < 1 point. The change just had statistical significance, instead of clinical significance. Additionally, we verified that this is the mRS observed at 1 month. Confirmation of long-term efficacy also depends on whether mRS changes significantly at 3 months.

Low-quality evidence and very low-quality evidence indicated that adjuvant acupuncture after thrombolysis might not increase the hemorrhagic conversion rate and incidence of other adverse reactions in patients within 48 h of AIS onset.

In addition to the aforementioned findings, we summarized the limitations of included SRs as follows: (1) All participants were diagnosed with AIS, but the definition of acute phase varied widely across SRs, including within 2 weeks of stroke onset, 4 weeks, 1 month, and 48 h. (2) Some SRs included 2 or more types of acupuncture, but there was no subgroup analysis based on different acupuncture therapies when performing meta-analyses. This clinical heterogeneity, to a certain extent, limited the generalizability of results.

### Strengths and limitations

As the first overview of acupuncture for AIS, this study provided a series of clinical evidence, graded according to the GRADE, contributing to decision-making; revealed prevalent problems in current SRs and RCTs; and made constructive suggestions for future researchers. However, there was an inevitable limitation. The quality evaluation is subjective, although we guarantee strict adherence to internationally recognized standards.

### Implications

To shorten the course of the disease, lengthen the duration of follow-up, increase sample sizes, and achieve blinding are the most critical implications for trialists. Firstly, the initiation time might be uniformly selected as the first 2 weeks after the onset of stroke. Generally, we define the acute phase as within 2 weeks (34). Moreover, the effect of acupuncture might be more significant during this period (16). Based on this premise, to further determine the optimum initiation time of acupuncture therapies, it is recommended to observe the difference in the efficacy of patients receiving acupuncture therapies within 6, 24, or 48 h of stroke onset. Secondly, given the potential advantages of acupuncture in improving disability rate and mortality after 3 or 6 months of follow-up (20), trialists should investigate endpoints, such as death, persistent disability, and recurrence of stroke, in more samples. Thirdly, trialists are suggested to blind outcome assessors, whereas sham acupuncture should be used with caution due to the underestimation of the acupuncture effect (35). As for placebo devices in acupuncture clinical trials, there was no definite evidence to support the blinding effects of these devices (36).

Reviewers of SRs should develop a protocol in advance to avoid performance bias; provide a complete list of excluded trials with exclusion reasons to avoid publication bias; fully address the heterogeneity and assess the impact of RoB on results to enhance credibility.

## Conclusion

During the acute and early recovery phases after AIS onset, acupuncture therapies improve the curative effect of current treatments, especially in restoring neurological function. Based on the current evidence, it is suggested to select acupuncture methods according to the stages after AIS onset. Patients within 48 h or 2 weeks might receive XNKQ Ac, and patients within 1 month might receive EA^1^, CA, or SA. Considering the certainty of evidence, trialists should verify the benefits of acupuncture therapies in the future.

## Data availability statement

The original contributions presented in the study are included in the article/Supplementary material, further inquiries can be directed to the corresponding authors.

## Author contributions

LW, XL, and YZ designed the study. JL provided help with methodology. XL and YZ made clinical suggestions and critically revised the manuscript. LW completed the introduction section. LW and ZX conducted study selection and data extraction. XC and GF performed AMSTAR 2 assessment. WQ and HL performed a GRADE assessment. SL and YL summarized the tables. LW and XC completed the manuscript writing. ZX helped with project supervision and language revisions. All authors contributed to the article and approved the submitted version.

## Funding

The study was funded by the Innovation Team and Talents Cultivation Program of the National Administration of Traditional Chinese Medicine (No. ZYYCXTD-C-202007); the project from the China Center for Evidence-Based Traditional Chinese Medicine (No. 2020YJSZX-3); the projects from China Academy of Chinese Medical Sciences (Nos. CI2021B006 and CI2021A01301); 2019 the State Administration of traditional Chinese medicine TCM evidence-based capacity building project (ZZ13-024-3) and the National TCM Leading Personnel Support Program [NATCM Personnel and Education Department (2018) No. 12].

## Conflict of interest

The authors declare that the research was conducted in the absence of any commercial or financial relationships that could be construed as a potential conflict of interest.

## Publisher's note

All claims expressed in this article are solely those of the authors and do not necessarily represent those of their affiliated organizations, or those of the publisher, the editors and the reviewers. Any product that may be evaluated in this article, or claim that may be made by its manufacturer, is not guaranteed or endorsed by the publisher.
