# Supplementary material for: An overview of the evidence to guide decision-making in acupuncture therapies for early recovery after acute ischemic stroke
Source: Front Neurol. 2022 Oct 13;13:1005819. doi: 10.3389/fneur.2022.1005819 (PMC9608668; doi:10.3389/fneur.2022.1005819)
Supplement: Supplementary file 1 [file Table_1.DOCX]

Supplementary Material

# Supplementary Tables

**Supplementary Table 1.** The search strategies for all databases.

| **The search strategy for MEDLINE** (1946 to 23 May 2022) | |
| --- | --- |
| **Number** | **Search terms** |
| #1 | cerebrovascular disorders/ or exp basal ganglia cerebrovascular disease/ or exp brain ischemia/ or exp brain infarction/ or exp cerebral infarction/ or exp carotid artery diseases/ or exp carotid artery thrombosis/ or exp intracranial arterial diseases/ or exp cerebral arterial diseases/ or exp "intracranial embolism and thrombosis"/ or exp stroke/ |
| #2 | (isch?emi$ adj6 (stroke$ or apoplex$ or cerebral vasc$ or cerebrovasc$ or cva or attack$)).tw. |
| #3 | ((brain or cerebr$ or cerebell$ or vertebrobasil$ or intracran$ or intracerebral or supratentorial or middle cerebral artery or MCA$ or anterior circulation or posterior circulation or basilar artery or vertebral artery or space-occupying) adj5 (isch? emi$ or infarct$ or thrombo$ or emboli$ or occlus$)).tw. |
| #4 | 1 or 2 or 3 |
| #5 | exp acupuncture therapy/ or exp electroacupuncture/ or exp meridians/ |
| #6 | exp Acupuncture/ |
| #7 | (acupuncture or electroacupuncture or meridian$ or needl$ or trigger point$).tw. |
| #8 | 5 or 6 or 7 |
| #9 | exp meta-analysis/ or exp "systematic review"/ |
| #10 | meta-analysis.pt. |
| #11 | (meta-analys$ or meta analys$).tw. |
| #12 | (systematic$ adj5 review$).tw. |
| #13 | 9 or 10 or 11 or 12 |
| #14 | 4 and 8 and 13 |
| **The search strategy for EMBASE** (1996 to 2022 Week 20) | |
| **Number** | **Search terms** |
| #1 | cerebrovascular disease/ or exp brain infarction/ or exp brain ischemia/ or exp cerebral artery disease/ or exp cerebrovascular accident/ or exp occlusive cerebrovascular disease/ |
| #2 | (isch?emi$ adj6 (stroke$ or apoplex$ or cerebral vasc$ or cerebrovasc$ or cva or attack$)).tw. |
| #3 | ((brain or cerebr$ or cerebell$ or vertebrobasil$ or intracran$ or intracerebral or supratentorial or middle cerebral artery or MCA$ or anterior circulation or posterior circulation or basilar artery or vertebral artery or space-occupying) adj5 (isch? emi$ or infarct$ or thrombo$ or emboli$ or occlus$)).tw. |
| #4 | 1 or 2 or 3 |
| #5 | exp acupuncture/ |
| #6 | (acupuncture or electroacupuncture or meridian$ or needl$ or trigger point$).tw. |
| #7 | 5 or 6 |
| #8 | exp "systematic review"/ |
| #9 | exp meta analysis/ |
| #10 | (meta-analys$ or meta analys$).tw. |
| #11 | 8 or 9 or 10 |
| #12 | 4 and 7 and 11 |
| **The search strategy for the Cochrane Library** | |
| **Number** | **Search terms** |
| #1 | MeSH descriptor: [Cerebrovascular Disorders] this term only |
| #2 | MeSH descriptor: [Basal Ganglia Cerebrovascular Disease] this term only |
| #3 | MeSH descriptor: [Brain Ischemia] explode all trees |
| #4 | MeSH descriptor: [Carotid Artery Diseases] this term only |
| #5 | MeSH descriptor: [Carotid Artery Thrombosis] this term only |
| #6 | MeSH descriptor: [Intracranial Arterial Diseases] this term only |
| #7 | MeSH descriptor: [Cerebral Arterial Diseases] this term only |
| #8 | MeSH descriptor: [Stroke] explode all trees |
| #9 | (isch?emi* near/6 (stroke* or apoplex* or cerebral vasc* or cerebrovasc* or cva or attack*)):ti,ab,kw |
| #10 | ((brain or cerebr* or cerebell* or vertebrobasil* or hemispher* or intracran* or intracerebral or infratentorial or supratentorial or middle cerebr* or mca* or anterior circulation) near/5 (isch?emi* or infarct* or thrombo* or emboli* or occlus* or hypoxi*)):ti,ab,kw |
| #11 | #1 or #2 or #3 or #4 or #5 or #6 or #7 or #8 or #9 or #10 |
| #12 | MeSH descriptor: [Acupuncture Therapy] explode all tree |
| #13 | MeSH descriptor: [Acupuncture] explode all trees |
| #14 | (acupuncture or electroacupuncture or meridian* or needl* or trigger point* or acupotom*) |
| #15 | #12 or #13 or #14 |
| #16 | MeSH descriptor: [Meta-Analysis as Topic] explode all trees |
| #17 | (systematic review):ti,ab,kw OR (Meta-Analysis):ti,ab,kw OR (meta analysis):ti,ab,kw OR (meta-analyses):ti,ab,kw |
| #18 | #16 or #17 |
| #19 | #11 and #15 and #18 |
| **The search strategy for CNKI** | |
| (SU = '缺血性脑卒中' or SU = '缺血性卒中' or SU = '缺血性脑中风' or SU = '缺血性中风' or SU = '缺血性脑血管病' or SU = '脑梗死' or SU = '脑梗' or SU = '脑栓塞' or SU = '脑缺血' or SU = '脑梗塞' or SU = '小脑梗塞' or SU = '脑干梗塞' or SU = '脑血栓') and (SU= '针刺' or SU = '电针' or SU = '醒脑开窍针' or SU = '针灸' or SU = '耳针' or SU = '体针' or SU = '舌针' or SU = '腹针' or SU = '头针' or SU = '火针') and (SU = '急性' or SU = '超早期' or SU = '进展性' or SU = '恶化性' or SU = '早期神经系统功能恶化') and (SU = '系统评价' or SU = 'Meta分析' or SU = '系统综述' or SU = '荟萃分析' or SU = '汇总分析' or SU = '集成分析' or SU = '二次分析' or SU = '衍生分析') | |
| **The search strategy for WanFang** | |
| 主题:("缺血性脑卒中" or "缺血性卒中"or"缺血性脑中风"or"缺血性中风"or"缺血性脑血管病"or"脑梗死"or"脑梗"or"脑栓塞"or"脑缺血"or"脑梗塞"or"小脑梗塞"or"脑干梗塞"or"脑血栓") and 主题:("针刺"or"电针"or"醒脑开窍针"or"针灸"or"耳针"or"体针"or"舌针"or"腹针"or"头针" or"火针") and 主题：("急性"or"超早期"or"进展性"or"恶化性"or"早期神经系统功能恶化") and 主题：("系统评价"or"Meta分析"or"系统综述"or"荟萃分析"or"汇总分析" or"集成分析"or"二次分析"or"衍生分析") | |
| **The search strategy for VIP** | |
| ((M=缺血性脑卒中 OR 缺血性卒中 OR 缺血性脑中风 OR 缺血性中风 OR 缺血性脑血管病 OR 脑梗死 OR 脑梗 OR 脑栓塞 OR 脑缺血 OR 脑梗塞 OR 小脑梗塞 OR 脑干梗塞 OR 脑血栓) OR (R=缺血性脑卒中 OR 缺血性卒中 OR 缺血性脑中风 OR 缺血性中风 OR 缺血性脑血管病 OR 脑梗死 OR 脑梗 OR 脑栓塞 OR 脑缺血 OR 脑梗塞 OR 小脑梗塞 OR 脑干梗塞 OR 脑血栓)) AND ((M=针刺 OR 电针 OR 醒脑开窍针 OR 针灸OR 耳针 OR 体针 OR 舌针 OR 腹针 OR 头针 OR 火针) OR (R=针刺 OR 电针 OR 醒脑开窍针 OR 针灸OR 耳针 OR 体针 OR 舌针 OR 腹针 OR 头针 OR 火针)) AND ((M=急性 OR 超早期 OR 进展性 OR 恶化性 OR 早期神经系统功能恶化) OR (R=急性 OR 超早期 OR 进展性 OR 恶化性 OR 早期神经系统功能恶化)) AND ((M=系统评价 OR Meta分析OR系统综述OR荟萃分析OR汇总分析OR集成分析OR二次分析OR衍生分析) OR (R=系统评价 OR Meta分析OR系统综述OR荟萃分析OR汇总分析OR集成分析OR二次分析OR衍生分析)) | |
| **The search strategy for CBM** | |
| #1 | "脑梗死"[不加权:扩展] |
| #2 | "缺血性脑卒中"[常用字段:智能] OR "缺血性卒中"[常用字段:智能] OR "缺血性脑中风"[常用字段:智能] OR "缺血性中风"[常用字段:智能] OR "缺血性脑血管病"[常用字段:智能] OR "脑梗"[常用字段:智能] OR "脑栓塞"[常用字段:智能] OR "脑缺血"[常用字段:智能] |
| #3 | "脑梗塞"[常用字段:智能] OR "小脑梗赛"[常用字段:智能] OR "脑干梗塞"[常用字段:智能] OR "脑血栓"[常用字段:智能] |
| #4 | "急性"[常用字段:智能] OR "超早期"[常用字段:智能] OR "进展性"[常用字段:智能] OR "恶化性"[常用字段:智能] OR "早期神经系统功能恶化"[常用字段:智能] |
| #5 | "针刺"[不加权:扩展] OR "针刺疗法"[不加权:扩展] |
| #6 | "针刺"[常用字段:智能] OR "电针"[常用字段:智能] OR "醒脑开窍针"[常用字段:智能] OR "针灸"[常用字段:智能] OR "耳针"[常用字段:智能] OR "体针"[常用字段:智能] OR "舌针"[常用字段:智能] OR "腹针"[常用字段:智能] OR "头针"[常用字段:智能] |
| #7 | "Meta分析"[不加权:扩展] |
| #8 | "系统评价"[常用字段:智能] OR "Meta分析"[常用字段:智能] OR "系统综述"[常用字段:智能] OR "荟萃分析"[常用字段:智能] OR "汇总分析"[常用字段:智能] OR "集成分析"[常用字段:智能] OR "二次分析"[常用字段:智能] OR "衍生分析"[常用字段:智能] |
| #9 | #1 OR #2 OR #3 |
| #10 | #5 OR #6 |
| #11 | #7 OR #8 |
| #12 | #9 AND #4 AND #10 AND #11 |

**Supplementary Table 2.** The list of excluded reports.

| **Report excluded** | **Reason** |
| --- | --- |
| Sang et al., 2022 | Ineligible course of disease |
| Xu et al., 2018 | Ineligible type of stroke |
| Ji et al., 2018 | Ineligible course of disease |
| Yang et al., 2015 | Duplicate data |
| Wang, 2015 | Ineligible interventions |

**References**

Sang, B., Deng, S., Zhai, J., Hao, T., Zhuo, B., Qin, C., et al. (2022). Does acupuncture therapy improve language function of patients with aphasia following ischemic stroke? A systematic review and meta-analysis. *Neurorehabilitation*. doi: 10.3233/NRE-220007

Xu, M., Li, D., and Zhang, S. (2018). Acupuncture for acute stroke. *Cochrane Db. Syst. Rev*.(3). doi: 10.1002/14651858.CD003317.pub3

Ji, J., Zhang, L., Zhang, X., and Liu, W. (2018). Systematic review of acupuncture at Du Mai point in the treatment of acute ischemic stroke. *World Journal of Integrated Traditional and Western Medicine*. 13(07), 900-903. doi: 10.13935/j.cnki.sjzx.180704

Yang, Z., Xie, J., Liu, Y., Miao, G., Wang, Y., Wu, S., et al. (2015). Systematic review of long-term Xingnao Kaiqiao needling effcacy in ischemic stroke treatment. *Neural Regen. Res*. 10(4), 583-8. doi: 10.4103/1673-5374.155431

Wang, Y. (2015). Systematic review and meta-analysis of the efficacy and safety of acupuncture and moxibustion in the treatment of acute ischemic stroke. Guangzhou University of Chinese Medicine.

**Supplementary Table 3.** The results of efficacy outcomes.

| Reviews | Interventions | Results of meta-analyses |
| --- | --- | --- |
| Shao et al. (2021) | GV Ac+CT vs. CT | **Activities of daily living:**  BI (5 RCTs, *MD* = 14.16, 95% *CI* 7.34 to 20.79, *P* < 0.0001, *I*^2^ = 88%); subgroup analysis (2 RCTs, *MD*_≤ 15d_ = 22.55, 95% *CI* 18.66 to 26.45, *P* < 0.00001, *I*^2^ = 0%; 3 RCTs, *MD*_> 15d_ = 8.80, 95% *CI* 5.87 to 11.72, *P* < 0.0001, *I*^2^ = 0%).  mRS (3 RCTs, *MD* = -0.63, 95% *CI* -0.95 to -0.32, *P* < 0.0001, *I*^2^ = 0%)  **Neurological deficit score:**  NIHSS (4 RCTs, *MD* = -1.18, 95% *CI* -1.52 to -0.83, *P* < 0.00001, *I*^2^ = 0%);  CSS/MESSS (3 RCTs, *MD* = -3.77, 95% *CI* -4.98 to -2.57, *P* < 0.00001, *I*^2^ = 0%). |
|  | GV Ac vs. CA | **Activities of daily living:**  BI (5 RCTs, *MD* = 8.27, 95% *CI* 4.29 to 12.26, *P* < 0.0001, *I*^2^ = 78%).  **Neurological deficit score:**  NIHSS (2 RCTs, *MD* = -1.32, 95% *CI* -2.18 to -0.47, *P* = 0.002, *I*^2^ = 75%);  CSS/MESSS (3 RCTs, *MD* = -4.63, 95% *CI* -5.91 to -3.35, *P* < 0.00001, *I*^2^ = 50%). |
| Zhang and Li (2018) | CA, EA^1^, EA^2^, SA or A&M+CT vs. CT | **Neurological deficit score:**  NIHSS (17 RCTs, *MD* = -1.86, 95% *CI* -2.06 to -1.66, *P* < 0.00001, *I*^2^ = 22%);  **Total effective rate:**  (26 RCTs, *OR* = 3.95, 95% *CI* 3.02 to 5.16, *P* < 0.00001, *I*^2^ = 0%). |
| Yang et al. (2017) | XNKQ Ac+CT vs. CT | **Disability rate:**  (3 RCTs, *RR* = 0.51, 95% *CI* 0.27 to 0.98, *P* = 0.04, *I*^2^ = 0%).  **Mortality:**  (3 RCTs, *RR* = 0.58, 95% *CI* 0.17 to 1.93, *P* = 0.37, *I*^2^ = 0%).  **Activities of daily living:**  BI (3 RCTs, *WMD* = 9.94, 95% *CI* -2.15 to 22.03, *P* = 0.11, *I*^2^ = 71%).  **Total effective rate:**  (8 RCTs, *RR* = 1.59, 95% *CI* 1.20 to 2.12, *P* = 0.001, *I*^2^ = 69%). |
|  | XNKQ Ac+CA vs. CA | **Activities of daily living:**  BI (1 RCT, *WMD* = 17.17, 95% *CI* 9.15 to 25.19, *P* < 0.0001).  **Total effective rate**:  (1 RCT, *RR* = 1.80, 95% *CI* 1.00 to 3.23, *P* = 0.05). |
| Liu et al. (2015) | EA^1^+CT vs. CT | **Activities of daily living:**  BI (10 RCTs, *SMD* = 0.72, 95% CI 0.35 to 1.08, *P* = 0.0001, *I*^2^ = 82%)  FMA (8 RCTs, *SMD* = 0.98, 95% CI 0.75 to 1.22, *P* < 0.00001, *I*^2^ = 36%)  **Neurological deficit score:**  NIHSS (6 RCTs, *SMD* = -0.81, 95% *CI* -1.14 to -0.49, *P* < 0.00001, *I*^2^ = 51%)  CSS (5 RCTs, *SMD* = -1.27, 95% *CI* -2.18 to -0.37, *P* = 0.006, *I*^2^ = 94%)  **Total effective rate:**  **(**6 RCTs, *RR* = 1.42, 95% *CI* 1.18 to 1.72, *P* = 0:0002, *I*^2^ = 16%) |
| Wang et al. (2012) | SA+CT vs. CT | **Neurological deficit score:**  MESSS **(**7 RCTs, *WMD* = −3.89, 95% *CI* −5.36 to −2.43, *P* < 0.00001, *I*^2^ = 57%)  **Total effective rate:**  (4 RCT, *RR* = 1.23, 95% *CI* 1.11 to 1.37, *P* < 0.01, *I*^2^ = 0%) |
| Zhang et al. (2009) | CA or EA^1^+CT vs. CT | **Rate of disability or death:**  (3 RCTs, *OR* = 0.59, 95% *CI* 0.31 to 1.13, *P* = 0.11, *I*^2^ = 0%)  **Neurological deficit score:**  SSS (3 RCTs, *WMD*=3.49, 95% *CI* 2.00 to 4.99, *P* < 0.00001, *I*^2^ = 81.9%) |

Ac, acupuncture; A&M, acupuncture and moxibustion; CA, conventional acupuncture; CT, conventional therapy; EA^1^, electroacupuncture; EA^2^, eye acupuncture; FA, foot acupuncture; GV, governor vessel; IVT, intravenous thrombolysis; SA, scalp acupuncture; XNKQ, Xingnao Kaiqiao needling method.
